# Structure of an In Situ Phosphorus-Doped Silicon Ultrathin Film Analyzed Using Second Harmonic Generation and Simplified Bond-Hyperpolarizability Model

**DOI:** 10.3390/nano12234307

**Published:** 2022-12-04

**Authors:** Wei-Ting Chen, Ting-Yu Yen, Yang-Hao Hung, Kuang-Yao Lo

**Affiliations:** Department of Physics, National Cheng Kung University, Tainan 70101, Taiwan

**Keywords:** nanophotonics, simplified bond-hyperpolarizability model, phosphorus-doped silicon ultrathin film, second harmonic generation

## Abstract

In fabricating advanced silicon (Si)-based metal–oxide semiconductors, the ability to inspect dopant distribution in Si ultrathin films (tens of nm) is crucial for monitoring the amount of dopant diffusion. Here, we perform an anisotropic reflective second harmonic generation (SHG) measurement to demonstrate the sensitivity of SHG to phosphorus (P) concentration within the range of $2.5\times 10^{17}$ to $1.6\times 10^{20}$ atoms/cm^3^. In addition, we propose an analysis method based on a simplified bond-hyperpolarizability model to interpret the results. The bond vector model that corresponds to the P vacancy clusters is built to calculate the SHG contribution from substitutionally incorporated P atoms. The effect of incorporating P into the Si lattice is reflected in the effective hyperpolarizability, lattice tilt, and deformation of this model. The fitting results of the intuitively defined coefficients exhibit a high correlation to the P concentration, indicating the potential of this model to resolve the properties in complex material compositions. Finally, a comparison with Fourier analysis is made to evaluate the advantages and disadvantages of this model. Combined anisotropic reflective SHG (Ani-RSHG) and the simplified bond-hyperpolarizability model (SBHM) can analyze the crystal structure of doped ultrathin films and provide a non-destructive nanophotonic way for in-line inspection.

## 1. Introduction

The semiconductor industry has adopted scaling innovations for generating complementary metal–oxide semiconductor (CMOS) logic technology, following Moore’s law [1,2]. CMOS scaling focuses on low-voltage, high-performance, and cost-effective processes to satisfy the requirements for high-efficiency calculations and high-end mobile applications. To overcome the integration issue and achieve low consumption, the shape of the CMOS has been changed from a planar structure to a 3D structure, such as fin field-effect transistors (FETs) [3,4]. Although device scaling approaches have reached an extreme level, suppressing short-channel effects by using fin FETs is still difficult. As a solution for 3D structure devices, FETs composed of multiple nanosheets with a gate-all-around (GAA) structure are good candidates for replacing fin FETs at the 5 nm technology node and beyond [5,6]. One of the most important achievements in 3D device manufacturing is the production of high and stable doping in the source and drain (S/D) region [1,3]. Moreover, the actual mobility of the nanosheets after the completed GAA fabrication, which is correlated to the out-diffusion S/D region, is also a key issue [6].

Ion implantation has played a required role as a doping method in the fabrication of larger-scale CMOS devices [4]. The skill of implantation offers a precise way to inject dopant species, such as boron (B) or phosphorous (P), in the required amount and distribution [7]. The drawback is that an annealing process is necessary to restructure the region damaged by implantation, inducing unavoidable dopant diffusion. This issue becomes more complicated as the critical dimension of metal–oxide semiconductor field-effect transistors evolve into the nanoscale. P-doped Si S/D that uses the in situ-doped epitaxy process has been developed because this process flow accepts a more accurate dopant concentration without additional thermal treatment [8,9]. However, in situ doping layers grown on the Si surface exhibit inherent defects that correlate with the growth condition and dopant density [5] due to a solubility limitation [10,11]. Investigations on P-doped Si films have suggested that donor–vacancy complexes can cause some of the doped P atoms to not emit free carriers; this phenomenon is called electrical deactivation [12,13,14]. Therefore, a nondestructive precise monitoring technique is essential for inspecting the crystal quality of the in situ-doped ultrathin film and optimizing it by fine-tuning the growth conditions and dopant concentration. In situ metrology benefits fabrication by predicting the film properties during the early stage, and the developed nondestructive method can also be used to analyze the dopant out-diffusion in nanosheets with an unavoidable thermal budget during fabrication.

Given that the in situ-doped ultrathin film is grown on the Si substrate, and its thickness is tens of nm, many limitations exist for crystalline analysis with nondestructive methods, particularly for nanosheets less than 10 nm. Although X-ray diffraction (XRD) can inspect the crystal structure distortion induced by the dopant, the recognizable lowest concentration is approximately 2% or 10^21^ atom/cm^3^ [9,12].

The high sensitivity of the reflective second harmonic generation (RSHG) to the symmetry breaking in the medium [15,16,17] has been utilized to analyze the restructuring and dopant correlation of ion-implanted Si [18,19]. The RSHG method exhibits a strong correlation between the surface dipole and the polarization of the incident light; hence, anisotropic RSHG (Ani-RSHG) spectra can realize their crystal symmetry and structure quality from anisotropic parameters [20,21]. Moreover, the Ani-RSHG method has been used to analyze the structure and stack properties of 2D materials, such as MoS_2_ and WS_2_, from the viewpoint of the surface electrical dipole, and it has become a standard analysis method [22,23]. The Ani-RSHG spectrum is a candidate for disclosing the structural evolution of a doped Si ultrathin film (DSUTF) that changes in symmetricity with the dopant concentration, although the crystalline structure of the DSUTF is imperfect. However, a model that can analyze the Ani-RSHG spectrum to determine detailed changes in the structure with various dopants remains lacking. Through the introduction of the simplified bond-hyperpolarizability model (SBHM), the number of independent second harmonic generation (SHG) susceptibility coefficients can be reduced by categorizing the bonds [24], allowing the analysis of the Ani-RSHG spectrum to be excited with the arbitrary polarization [24,25]. Furthermore, by comparing with Fourier analysis, which is conventionally used for the quantitative depiction of Ani-RSHG spectra [26,27], the fitting for the parameters defined on the basis of the SBHM makes the interpretation more intuitive.

In the current work, we propose a model for analyzing the crystalline properties of P-DSUTFs with different dopant concentrations. In addition to the effect of the dopant on the Si lattice, the SHG contribution of the dopant itself is modeled in an analogical manner to the electrostatic dipole moment. A substitutional P site is modeled by interpreting a P-vacancy (P-V) cluster into a vacancy cell with Si-vacancy (Si-V) and P-V bonds. To validate the proposed model, we performed Ani-RSHG experiments on a Si ultrathin film doped with various P concentrations. The measurements were performed in a vacuum chamber to avoid the additional electric field-induced SHG (EFISHG) caused by the surface charging effect [28,29]. The modeling of Ani-RSHG with the SBHM provides satisfactory results for in situ P-DSUTFs, suggesting the high potential of this modeling approach with the SBHM in characterizing DSUTFs.

## 2. Theory

### 2.1. SBHM

**Figure 1:**
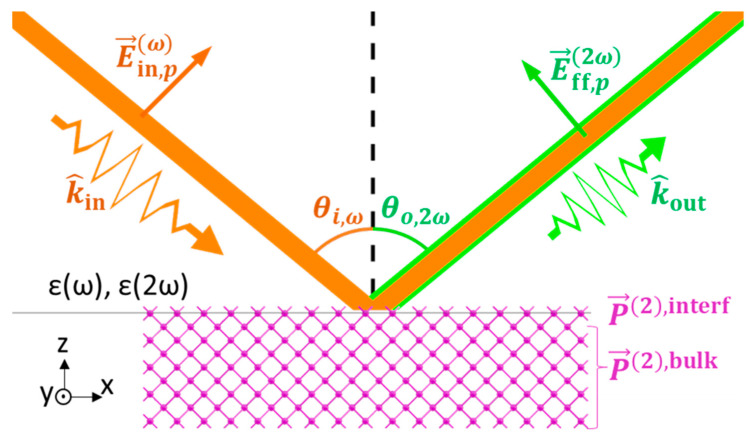
Geometry of fundamental and RSHG waves in the material of interest.

With the fundamental supposition that SHG originates only from the anharmonic motion of electrons along the bonds, the SBHM can predict the Ani-RSHG spectrum of crystalline structures by assuming the contribution of individual bonds. The geometry and unit vectors for the propagating fields are illustrated in Figure 1. In general, the induced SHG polarization density can be written in the following form:

(1) $${\vec{P}}_{eff}=a{\vec{P}}^{\left(2\right),\mathrm{interf}}\delta \left(z\right)+{\vec{P}}^{\left(2\right),\mathrm{bulk}}\theta \left(-z\right),$$

where a is the lattice constant, δ is the Dirac delta function, θ is the Heaviside step function, $\left|z\right|$ is the depth from the interface of the material of interest, and ${\vec{P}}^{\left(2\right),\mathrm{surf}}$ and ${\vec{P}}^{\left(2\right),\mathrm{bulk}}$ are the surface and bulk SHG polarization densities, respectively [30]. Si is a centrosymmetric lattice wherein bulk dipolar contribution is forbidden; thus, the dominant contribution is dipolar SHG at the interfaces and quadrupolar SHG in the bulk [31]. Although quadrupolar SHG is a higher-order term relative to a dipolar one, the contributions of the interfacial dipolar and bulk quadrupolar are conventionally considered because the bulk has more layers than the surface. In a doped Si thin film, however, the sites occupied by dopant atoms exhibit a broken symmetry, and thus, they irradiate the dipolar second harmonic (SH) waves. Here, we consider the dipolar contribution from the Si lattice on the surface, the dipolar contribution from the dopant sites, and the quadrupolar contribution from the centrosymmetric Si lattice in the bulk. Tilting and deformation are applied to the structures because incorporating P into Si may reduce the uniformity of the lattice structure [8,12].

The SHG polarization of a bond induced by the incident wave is determined by the relative direction of the bond and polarization. At the SiO_2_/Si interface and defect sites, the dominant SHG source is the dipolar terms, which can be modeled in the SBHM via [17,32,33,34]:

(2) $${\vec{P}}_{D}^{\left(2\right),\mathrm{interf}}=\chi_{D}^{\left(2\right)}\cdot \cdot {\vec{E}}_{\mathrm{in}}{\vec{E}}_{\mathrm{in}}=\frac{1}{V}\sum_{j=1}^{n}\left(\alpha_{j}^{\left(D\right)}{\hat{b}}_{j}{\hat{b}}_{j}{\hat{b}}_{j}\right)\cdot \cdot {\vec{E}}_{\mathrm{in}}{\vec{E}}_{\mathrm{in}},$$

whereas the quadrupolar term that dominates in the bulk can written in the following form [33,35]:

(3) $${\vec{P}}_{Q}^{\left(2\right),\mathrm{bulk}}=\chi_{Q}^{\left(2\right)}\cdot \cdot \cdot {\vec{E}}_{\mathrm{in}}{\vec{\nabla }}_{b}{\vec{E}}_{\mathrm{in}}=\frac{1}{V}\alpha_{\mathrm{bulk}}^{\left(Q\right)}\sum_{j=1}^{n}\left({\hat{b}}_{j}{\hat{b}}_{j}{\hat{b}}_{j}{\hat{b}}_{j}\right)\cdot \cdot \cdot {\vec{E}}_{\mathrm{in}}{\vec{\nabla }}_{b}{\vec{E}}_{\mathrm{in}},$$

where $\chi_{D}^{\left(2\right)}$ and $\chi_{Q}^{\left(2\right)}$ are the third and fourth rank susceptibility tensors, respectively; ${\vec{E}}_{\mathrm{in}}$ and ${\vec{\nabla }}_{b}$ are the E field of the incident wave and its gradient, respectively; V is the volume; n is the total number of bonds inside the considered unit cells; ${\hat{b}}_{j}$ is the bond unit vector; $\alpha_{j}^{\left(D\right)}$ is the dipolar SHG hyperpolarizability that corresponds to ${\hat{b}}_{j}$, and $\alpha_{\mathrm{bulk}}^{\left(Q\right)}$ is the quadrupolar SHG hyperpolarizability that is consistent with all the bonds in the bulk term. The gradient of the E field along the direction of the incident wave, ${\hat{k}}_{\mathrm{in}}$, takes the form

(4) $${\vec{\nabla }}_{b}=-iC_{\mathrm{grad}}{\hat{k}}_{\mathrm{in}},$$

where $C_{\mathrm{grad}}$ is a complex fitting number [24,25]. In the subsequent fitting, we adopted the value fitted out for the Si in the previous work [24,25]. The E field of the incident wave is determined by the angle of incidence, θ, and the polarization, ϕ, (0 for *s*- and 90 for *p*-polarization), such that

(5) $${\vec{E}}_{\mathrm{in}}=\left(\begin{array}{c} F_{\omega ,p}\cos\theta_{i,\omega }\sin\phi \\ F_{\omega ,s}\cos\phi \\ F_{\omega ,p}\sin\theta_{i,\omega }\sin\phi \end{array}\right)E_{0},$$

where $E_{0}$ is the field amplitude in the air, and $F_{\omega ,p}$ and $F_{\omega ,s}$ are the Fresnel transmission coefficients for the *p*- and *s*-polarization of the interfaces between the air and the SHG source, respectively. For each SHG polarization, ${\vec{P}}_{2\omega }$, obtained in Equations (2) and (3), a far-field SH radiation of *pol*-polarization can be predicted as

(6) $${\vec{E}}_{\mathrm{ff},pol}^{\left(2\omega \right)}\propto F_{2\omega ,pol}\left[\left(\bar{I}-{\hat{k}}_{\mathrm{out}}{\hat{k}}_{\mathrm{out}}\right)\cdot {\vec{P}}_{2\omega }\right],\;pol=p,s,$$

where $F_{2\omega ,pol}$ is the Fresnel transmission coefficient that corresponds to the electromagnetic wave at a frequency of 2*ω* and *pol*-polarization, $\bar{I}$ is the identical matrix, and ${\hat{k}}_{\mathrm{out}}$ is the unit vector in the direction of the measured SH radiation. The intensity of the measured SHG can be obtained as the square of the coherent superposition of the fields from all the sources. 

### 2.2. Bulk Quadrupolar SHG from Si(100)

**Figure 2:**
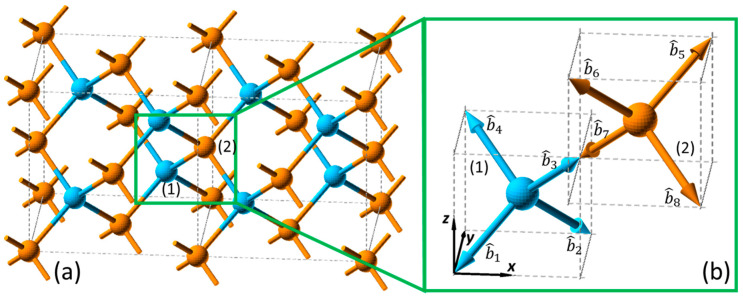
(**a**) Bond and (**b**) bond vector structure of Si(100) in the SBHM.

In the bond structure of Si(100) in the diamond structure shown in Figure 2a, the corresponding bond vectors can be determined as the unit vectors along the direction from the central atoms to the four bonding atoms. With the central atom at Position (1), the four bond vectors can be found as follows:

(7) $$\begin{array}{cc} {\hat{b}}_{1}=\left(\begin{array}{c} -\frac{1}{\sqrt{2}}\sin\frac{\beta }{2}\\ -\frac{1}{\sqrt{2}}\sin\frac{\beta }{2}\\ -\cos\frac{\beta }{2} \end{array}\right) & {\hat{b}}_{2}=\left(\begin{array}{c} \frac{1}{\sqrt{2}}\sin\frac{\beta }{2}\\ \frac{1}{\sqrt{2}}\sin\frac{\beta }{2}\\ -\cos\frac{\beta }{2} \end{array}\right)\\ {\hat{b}}_{3}=\left(\begin{array}{c} -\frac{1}{\sqrt{2}}\sin\frac{\beta }{2}\\ \frac{1}{\sqrt{2}}\sin\frac{\beta }{2}\\ \cos\frac{\beta }{2} \end{array}\right) & {\hat{b}}_{4}=\left(\begin{array}{c} \frac{1}{\sqrt{2}}\sin\frac{\beta }{2}\\ -\frac{1}{\sqrt{2}}\sin\frac{\beta }{2}\\ \cos\frac{\beta }{2} \end{array}\right) \end{array},$$

Meanwhile, with the central atom at Position (2), another four bond vectors can be found at the right opposite directions, such that

(8) $$\begin{array}{cc} \begin{array}{cc} {\hat{b}}_{5}=-{\hat{b}}_{1} & {\hat{b}}_{6}=-{\hat{b}}_{2} \end{array} & \begin{array}{cc} {\hat{b}}_{7}=-{\hat{b}}_{3} & {\hat{b}}_{8}=-{\hat{b}}_{4} \end{array} \end{array},$$

where $\beta =2\arccos\left(1/\sqrt{3}\right)\approx 109.4{}^{\circ}$ is the angle between each bond, as shown in Figure 2b.

In the bulk of the Si(100) lattices, the dipolar contribution is not allowed, and quadrupolar SHG polarization can be modeled by Equation (3) with the eight bonds described in Equations (7) and (8), and the bulk quadrupolar hyperpolarizability, $\alpha_{\mathrm{bulk}}^{\left(Q\right)}$, as a fitting parameter. Given that the same type of bond is of consideration, the quadrupolar response of each bond should be consistent from a microscopic viewpoint. Therefore, the changes in these terms reflect the macroscopic uniformity of the lattice structure, which is indicated by $\alpha_{\mathrm{bulk}}^{\left(Q\right)}$ in the fitting model.

### Si Lattice Affected by Doping

As reported, the presence of the P dopant in the Si lattice affects the lattice constant and uniformity [8,12]; accordingly, a fitting parameter is designated to represent the effect of the dopant on the Si(100) lattice structure. 

In a P-doped Si thin film on the Si substrate, the constraint of the Si substrate restricts the variation of the lattice constant in the in-plane dimensions, leading to a uniaxial extension in the dimension normal to the film–substrate interface [12,36]. In the SBHM fitting model, the deformation ratio, D, is defined as the change ratio of size along the axial direction, and it performs on the bond vectors by

(9) $${\hat{b}}_{j}^{\prime }=\frac{{\vec{b}}_{j}^{\prime }}{\left|{\vec{b}}_{j}^{\prime }\right|},\;{\vec{b}}_{j}^{\prime }=D\hat{s}\left(\hat{s}\cdot {\hat{b}}_{j}\right)+{\hat{b}}_{j},$$

where ${\hat{b}}_{j}^{\prime }$ is the unit vector that corresponds to the deformed bond, ${\hat{b}}_{j}$ is the original bond vector, and $\hat{s}$ is the axis of deformation. The axis of deformation is not consistent along the sample surface normal due to the degradation of the lattice uniformity.

When the incorporated dopant is sufficient to reduce the uniformity of the lattice structure, the facet orientations are expected to deviate slightly, for which an effective orientation can be determined within the range of measurement. Defined as the angle between the effective orientation of the [001] facet and the sample surface normal (and the axis of the azimuthal rotation in the Ani-RSHG measurement), the effective tilt angle, $\theta_{t}$, in the fitting model depicts the degree to which the lattice is disoriented by the involvement of the dopant. Notably, we considered a macroscopic measurement of the lattices that are randomly misoriented by the incorporation of the dopant in the current work, resulting in lattices with a variety of misorientation angles being measured at once, and thus, the fitting parameter $\theta_{t}$ cannot be defined as the angle of misorientation.

### 2.3. Interfacial Dipolar SHG from Si(100)

Following the bulk lattice structure, the Si lattices at the interface share the same bond vectors with the bulk Si lattices. However, dipolar SHG is allowed, and different dipolar hyperpolarizabilities are assigned to the bond vectors in accordance with the interface, because the symmetry is broken by the interface. At the Si atomic layer adjacent to the native SiO_2_, the hyperpolarizabilities of the bonds toward the SiO_2_ layer and the ones toward the inside of the bulk Si are different. For the bond vector structure expressed by Equations (7) and (8), dipolar hyperpolarizabilities $\alpha_{u}^{\left(D\right)}$ and $\alpha_{d}^{\left(D\right)}$ are assigned to the upward (${\hat{b}}_{3},{\hat{b}}_{4},{\hat{b}}_{5},{\hat{b}}_{6}$) and downward (${\hat{b}}_{1},{\hat{b}}_{2},{\hat{b}}_{7},{\hat{b}}_{8}$) bonds, respectively, depending on the sign of the z-component of the bond vectors, ${\hat{b}}_{j,z}$. However, the dipolar SHG intensity is isotropic to the azimuthal rotation because of the C_4v_ symmetry of this bond vector structure [37], and thus, the net contribution from an interface can be evaluated by the difference of the up and down hyperpolarizabilities in the following form:

(10) $${\vec{P}}_{D}^{\left(2\right),\mathrm{interf}}=\frac{1}{V}\alpha_{\mathrm{interf}}^{\left(D\right)}\sum_{j=3}^{6}\left({\hat{b}}_{j}{\hat{b}}_{j}{\hat{b}}_{j}\right)\cdot \cdot {\vec{E}}_{in}{\vec{E}}_{in},\;\alpha_{\mathrm{interf}}^{\left(D\right)}=\alpha_{u}^{\left(D\right)}-\alpha_{d}^{\left(D\right)},$$

where $\alpha_{\mathrm{interf}}^{\left(D\right)}$ is the effective hyperpolarizability of the interface [30,38]. Notably, only the upward bonds $\left(j=3\sim 6\right)$ are included in the summation that corresponds to the definition of $\alpha_{\mathrm{interf}}^{\left(D\right)}$. 

### 2.4. Dipolar SHG from the Dopant Site

**Figure 3:**
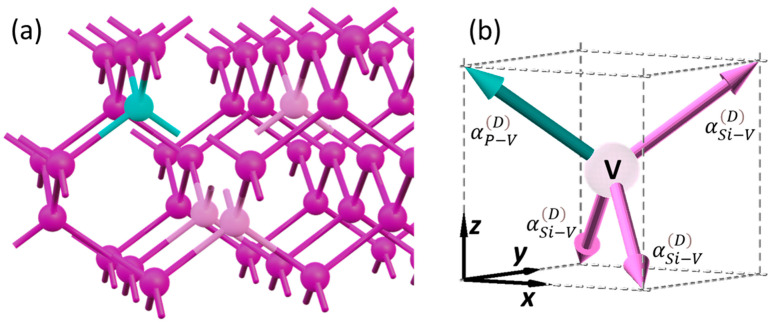
(**a**) The structure of a P_1_V cluster, where the Si and P atoms are colored in pink and cyan, respectively, and the P atoms neighboring the vacancy are colored in light pink. (**b**) A vacancy cell with the corresponding bond vector structure.

**Figure 4:**
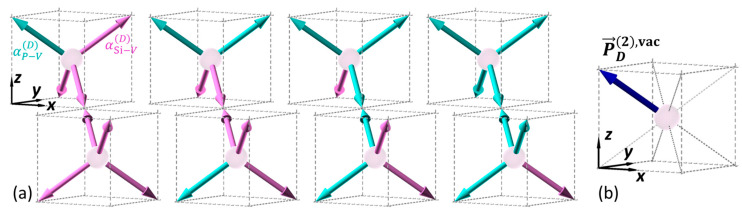
(**a**) Cases of the bond vector structure in a vacancy cell pair that provides the same (**b**) dipolar SHG polarization along a bond.

In the as-grown Si:P samples, most of the in situ-doped P atoms are incorporated into the substitutional Si sites. Previous experimental and theoretical studies have suggested that the substitutional P in Si forms P-V clusters [12,13,39], which exhibit dipole moments depending on the number and position of the P [14]. Similar to the electrostatic dipole moment, the hyperpolarizabilities of the P-V $\alpha_{P-V}^{\left(D\right)}$ and Si-V $\alpha_{Si-V}^{\left(D\right)}$ bonds can be determined by representing the behavior of the electrons between the vacancy and the adjacent atoms, because SHG polarization originates from the anharmonic motion of the electrons along the bonds. Furthermore, the electronic density calculated using a Vienna Ab initio Simulation Package (VASP) has shown the presence of vacancy bonds and the difference in density around P and Si, suggesting that $\alpha_{P-V}^{\left(D\right)}$ and $\alpha_{Si-V}^{\left(D\right)}$ should have different values [13]. Thus, a P-V cluster in Si(100) can be interpreted as a tetragonal bond structure that centers at a vacancy bonding to Si (colored in light pink) or P (cyan) atoms, as shown in Figure 3 [14]. The difference in the bonding atoms leads to a non-centrosymmetric structure, where dipolar SHG polarization is allowed. By considering a pair of bond vector cells with vacancy centers at Positions (1) and (2) in Figure 2 (referred to as the vacancy cell pair, hereafter), the same bond vectors described by Equations (7) and (8) can also correspond to this structure. Similar to the interfacial dipolar terms, the SHG polarizations sourced from the bond vectors in the opposite direction vanish if the same hyperpolarizability is assigned to them. The possible number of P incorporated into a P_n_V cluster ranges from one to four. For simplicity, we only considered the cases wherein the net polarization of a vacancy cell pair was along one of the eight bonds. This condition can be satisfied in vacancy cell pairs with only one pair of bond vectors in the opposite direction with unequal hyperpolarizability, namely,

(11) $$\alpha_{5}^{\left(D\right)}\neq \alpha_{1}^{\left(D\right)},\;\alpha_{6}^{\left(D\right)}\neq \alpha_{2}^{\left(D\right)},\;\alpha_{7}^{\left(D\right)}\neq \alpha_{3}^{\left(D\right)}\;\mathrm{or}\;\alpha_{8}^{\left(D\right)}\neq \alpha_{4}^{\left(D\right)},$$

where $\alpha_{j=1\sim 8}^{\left(D\right)}$ is $\alpha_{P-V}^{\left(D\right)}$ or $\alpha_{Si-V}^{\left(D\right)}$. With this configuration, the contribution from the dopant sites only originates from a pair of bond vector cells with asymmetric bond pairs and effective hyperpolarizability, i.e.,

(12) $$\alpha_{\mathrm{vac}}^{\left(D\right)}=\alpha_{P-V}^{\left(D\right)}-\alpha_{Si-V}^{\left(D\right)},$$

is assigned to the vector that corresponds to the PV bond. Four examples of the vacancy cells satisfying this condition are illustrated in Figure 4a, where the pink and cyan arrows correspond to the Si-V and P-V bonds, respectively. As shown in Figure 4b, the net dipolar SHG polarization of each vacancy cell pair shown in Figure 4a is along the bond vector ${\hat{b}}_{6}$ (defined in Equation (8)). The fitted $\alpha_{\mathrm{vac}}^{\left(D\right)}$ value indicates the density of such P_n_V clusters in the Si thin film.

## 3. Materials and Methods

### 3.1. Measurement of Ani-RSHG Spectrum

**Figure 5:**
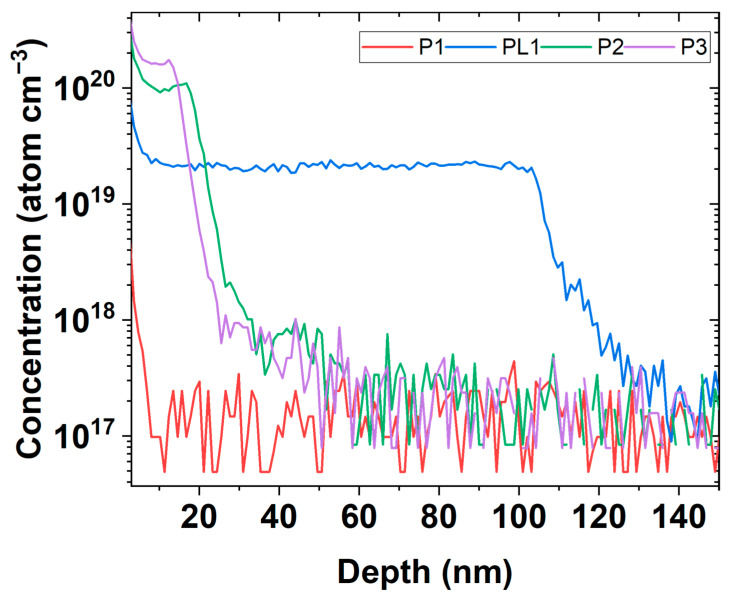
SIMS of the P-doped DSUTF samples.

Samples of DSUTFs were grown on a Si(100) substrate via chemical vapor deposition (CVD). P1, P2, and P3 denote samples with a thickness of 20 nm and P concentrations of approximately $2.5\times 10^{17}$, $9.8\times 10^{19}$, and $1.6\times 10^{20}$ atoms/cm^3^, respectively. Besides, a P-doped Si ultrathin film thickness of 100 nm and a P concentration of $2.3\times 10^{19}$ atoms/cm^3^, named PL1, were provided for further comparison and study. The actual dopant concentration and thickness of these samples were measured via secondary ion mass spectroscopy (SIMS) at the National Synchrotron Radiation Research Center in Taiwan (Figure 5). The lower limit of the measurable P concentration with this SIMS is determined to be $1.5\times 10^{17}$ atoms/cm^3^ by the mean value measured at a depth of *z* > 100 nm in the samples P2 and P3.

**Figure 6:**
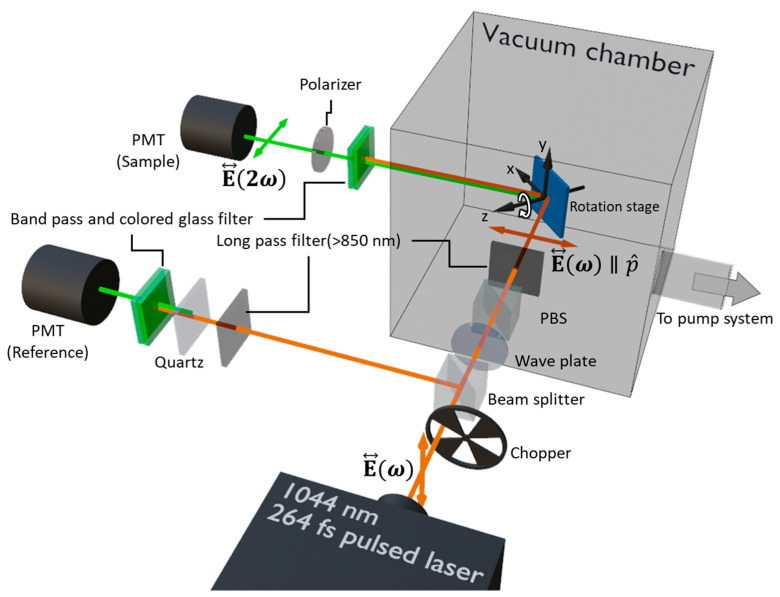
Experiment setup for Ani-RSHG measurement in a vacuum.

The Ani-RSHG experiment system is shown in Figure 6. The light source of the Ani-RSHG experiment was a pulsed laser (femtoTRAIN IC-1040-2000, Newport Corporation, Irvine, CA, USA) with a wavelength of 1044 nm, pulse duration of 264 fs, and repetition frequency of 20.86 MHz. The irradiated spot area was $1.7\times 10^{-4}\;{\mathrm{cm}}^{2}$, the average power was 12 kW/cm^2^, and the peak power density irradiated onto the samples was 2.2 GW/cm^2^, which was below the damage threshold of DSUTF. In order to describe the optical pass of the laser beam in this experiment, a Cartesian coordinate system (x-y-z) was built on the plane of the rotation stage, as indicated in Figure 6. This coordinate system on the plane of the rotation stage is correlated to the one shown in Figure 1. To measure the anisotropic nonlinear optical properties of the sample, the rotation stage was used to rotate the loaded sample by 2.25° per step about the *z*-axis during the measurement of the Ani-RSHG spectra. The pulsed laser with a linear polarization, $\overset{↔}{E}\left(\omega \right)$, passed through first the chopper (SR450; Stanford Research Systems, Sunnyvale, CA, USA) with a speed of 100 rounds per second, and then a cubic beam splitter (CCM1-BS014; Thorlabs, Newton, NJ, USA). The pulsed laser beam was split into the transmitted and reflected parts after the beam splitter cube. The transmitted part of the beam subsequently passed through a 1044-nm half-waveplate (WPQSM05-1053, Thorlabs, Newton, NJ, USA), a polarizing beam splitter cube (denoted PBS in Figure 6, CCM1-PBS253; Thorlabs, Newton, NJ, USA), and a long pass filter (850 nm Longpass; Thorlabs, Newton, NJ, USA) before reaching the sample, which was fixed on the plane of the rotation stage. The combination of the half-wave plate and the polarizing beam splitter was used for the laser polarization and intensity modulation. The extinction ratio of the *p*-polarized fundamental beam reaching the sample was approximately 400. The long pass filter was used to block out the noise light in the background. While an Ani-RSHG experiment was performed in the vacuum chamber, the pressure of the chamber was kept below 10^−5^ torr to avoid the charge effect due to oxygen gas adsorption [28,29].

The RSHG signal was generated from the DSUTF while the *p*-polarized fundamental beam irradiated on the DSUTF. After exiting the vacuum chamber, the RSHG beam passed through a filter set and a *p*-polarizer before entering the photomultiplier tube (denoted PMT (Sample) in Figure 6) (R1527P; Hamamatsu Photonics, Shizuoka, Japan), which recorded the *pp*-polarized RSHG signal. The filter set was composed of a bandpass filter and a colored glass filter (colored Glass FBG 39; Thorlabs, Newton, NJ, USA, and bandpass filter 520 nm; Edmond, OK, USA).

The reflected part of the beam from the beam splitter passed through a long pass filter, quartz, and a filter set before entering the PMT for the reference signal (denoted PMT (Reference) in Figure 6) (R1527P; Hamamatsu Photonics, Shizuoka, Japan). The quartz was used as a stable source of the SHG signal to monitor the laser power fluctuation. Ani-RSHG spectra were obtained by recording the raw data value recorded from the PMT (Sample) divided by that recorded from the PMT (Reference). In this work, Ani-RSHG spectra were recorded and analyzed by a lock-in amplifier system (SR830; Stanford Research Systems, Sunnyvale, CA, USA).

### 3.2. Process of Data-Fitting Based on the SBHM

As modeled in the *Theory* section, the Ani-RSHG spectra measure the intensity of the superposition of the considered contributions, the Si(100) interfacial dipole, Si(100) bulk quadrupole, and dopant-vacancy cite dipole, i.e.,

(13) $$I_{\mathrm{sum},\;pp}^{\left(2\omega \right)}={\left|{\vec{E}}_{\mathrm{sum},pp}^{\left(2\omega \right)}\right|}^{2}\propto {\left|\left(\bar{I}-{\hat{k}}_{\mathrm{out}}{\hat{k}}_{\mathrm{out}}\right)\cdot {\vec{P}}_{\mathrm{sum},\;2\omega }\right|}^{2},$$

where

(14) $${\vec{P}}_{\mathrm{sum},\;2\omega }={\vec{P}}_{D}^{\left(2\right),\mathrm{interf}}+{\vec{P}}_{Q}^{\left(2\right),\mathrm{bulk}}+{\vec{P}}_{D}^{\left(2\right),\mathrm{vac}}.$$

The fitting of the simulated spectra to the measured spectra is based on the theoretical model, i.e., Equation (13). In the fitting, trial parameters were introduced to test the system and iterate to find the appropriate values to fit the measured Ani-RSHG spectra. The choice of the trial parameters was based on the interpretation of the possible effects caused by changes in the P concentration in terms of the lattice structure. The iteration of the trial parameters will be converged with the value that minimizes the difference between the theoretical and measured spectra. If a consistent convergence is not achieved, the model should be adjusted, and the fitting process should be repeated.

The incorporated trial parameters include characteristic and orientational (or auxiliary) parameters. Characteristic parameters include the effective hyperpolarizabilities for SHG contributions from the bulk, $\alpha_{\mathrm{bulk}}^{\left(Q\right)}$, the interface, $\alpha_{\mathrm{interf}}^{\left(D\right)}$, the vacancy sites, $\alpha_{\mathrm{vac}}^{\left(D\right)}$, the lattice tilt angle, $\theta_{t}$, and the deformation ratio, *D*, whose interpretations were described in the theory section. Auxiliary parameters include the azimuthal lattice direction, deformation direction, $\hat{s}$, and direction of the net dipolar SHG polarization of the defect sites. These auxiliary parameters are subject to the relation between the sample coordinates (the plane of the rotation stage) and the lattice coordinates (Si(100)). There are no noticeable implications for the material properties.

## 4. Results and Discussion

**Figure 7:**
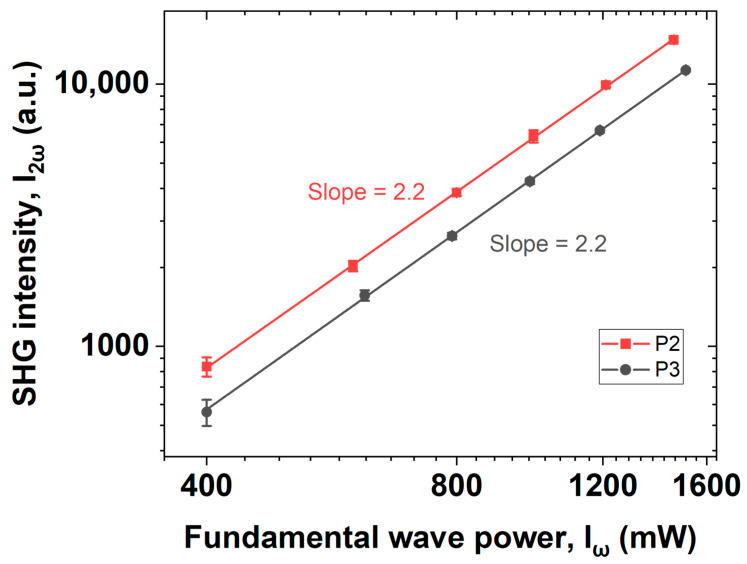
Second harmonic intensity depends quadratically on the fundamental wave power.

First, we examined the SHG phenomenon by observing the power law dependency on two samples. Figure 7 shows the second-order dependence of the doubled-frequency signal intensity on the power of the fundamental wave, which confirms the nonlinear nature of the SHG mechanism.

**Figure 8:**
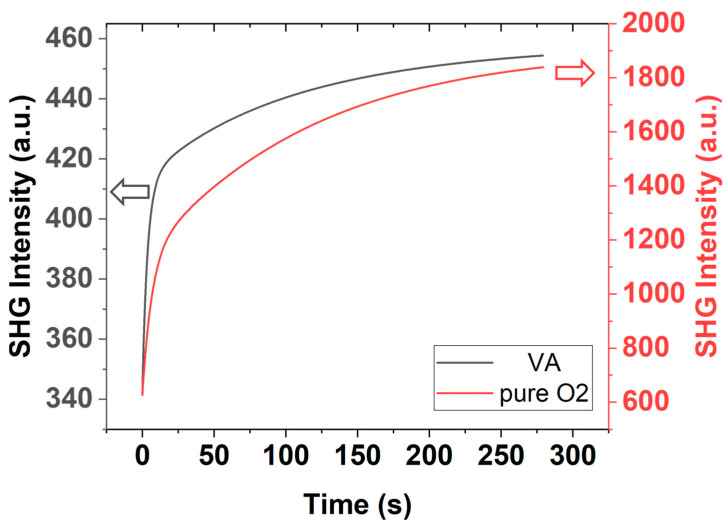
Time-dependent SHG of P1 in a vacuum (left *y*-axis scale) and pure O_2_ gas (right *y*-axis scale).

Due to the photon-induced charging effect, in which electrons are trapped by the absorbed gas molecules, an additional EFISHG contribution rises slowly but extends for a long time under continuous exposure to the pulsed laser of the fundamental light. This phenomenon can be observed by comparing the time-dependent SHG of P1 measured under a vacuum of 10^−5^ torr and under 2 torr of oxygen pressure (Figure 8). EFISHG, induced by the irradiation of the fundamental light, will violate the analysis of the crystalline property of the test samples in ambient gas; therefore, Ani-RSHG measurements in the following were performed in a vacuum chamber with a pressure of 10^−5^ torr. This measurement is time-independent and free from the violation of EFISHG, as shown in Figure 8.

**Figure 9:**
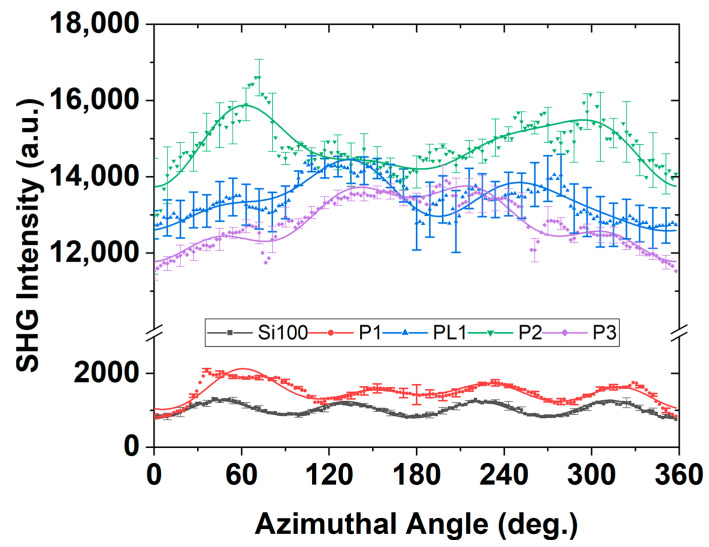
*pp*-polarized Ani-RSHG spectrum of P-doped Si(100).

**Figure 10:**
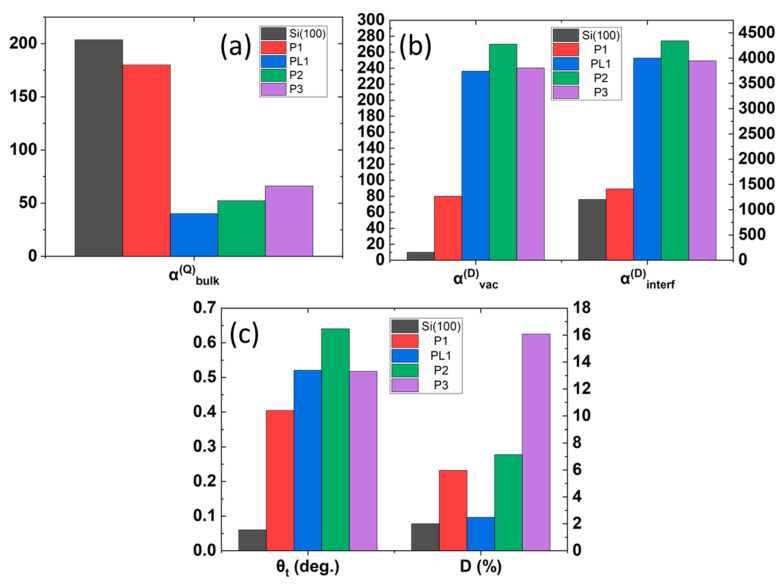
Fitted the SBHM parameters of the P-doped Si(100) Ani-RSHG spectrum. Fitted parameters include the effective hyperpolarizabilities of (**a**) bulk quadrupolar, $\alpha_{\mathrm{bulk}}^{\left(Q\right)}$, (**b**) vacancy dipolar, $\alpha_{\mathrm{vac}}^{\left(D\right)}$, and interface dipolar, $\alpha_{\mathrm{interf}}^{\left(D\right)}$, SHG, (**c**) the lattice tilt angle, $\theta_{t}$, and the deformation ratio, *D*.

The Ani-RSHG spectra and the analyzed coefficients for Samples P1~P3 with the reference of the Si(100) substrate are shown in Figure 9 and Figure 10, respectively.

In Figure 9, the mean value of several Ani-RSHG measurements is shown in dots, and the solid lines are the fitted curves of the theoretically simulated spectra. It is obvious that the fluctuation of the SHG signal is getting higher while the P dopant concentration increases because the uniformity of the film surface decreases as the dopant concentration increases. Additionally, the Ani-RSHG spectra of the thicker P-DSUTF, PL1, were calibrated for the effect of the thickness for comparison (see Section 4.1). The samples were arranged in ascending order of P concentration. The changes in the composition and lattice quality among the samples with an increasing P concentration were revealed by inspecting the changes in the key parameters shown in Figure 10.

As described in Section 2.2, changes in $\alpha_{\mathrm{bulk}}^{\left(Q\right)}$ indicate the uniformity of the lattice structure. In Figure 10a, the $\alpha_{\mathrm{bulk}}^{\left(Q\right)}$ value is reduced as the P concentration increases, suggesting that the addition of P degrades the crystalline order of the Si lattice. Following the discussion in Section 2.4, $\alpha_{\mathrm{vac}}^{\left(D\right)}$ intuitively responds to the density of the P_n_V cluster in a positive correlation. As shown in Figure 10b, the $\alpha_{\mathrm{vac}}^{\left(D\right)}$ increases with the doping concentration. Similar behavior is also observed in the effective lattice tilt angle, $\theta_{t}$, shown in Figure 10b, indicating an increasing degree of misorientation of the lattices caused by the increase of the incorporated P dopant. Whether one considers the precipitation of the dopants toward the SiO_2_/Si interface or simply the percentage of P concentration as the probability of the P atoms appearing in the substitutional Si sites at the interface, it is expected that the change in the doping concentration affects the net dipolar SHG contribution from the SiO_2_/Si interface, which can be observed on the $\alpha_{\mathrm{interf}}^{\left(D\right)}$ in Figure 10b. In the trends of the $\alpha_{\mathrm{bulk}}^{\left(Q\right)}$, $\alpha_{\mathrm{vac}}^{\left(D\right)}$, and $\theta_{t}$ (in Figure 10c) with an increasing concentration, P3 is demonstrated to be a common exception. Considering the lattice mismatch of 9.0% between the Si and P [40,41], the unrealistically high value of the deformation ratio, *D*, of P3 shown in Figure 10c suggests an explanation for this reverse trend, i.e., that the lattice structure severely deviates from the Si(100) matrix, such that the proposed dopant–vacancy model is no longer sufficient to predict the Ani-RSHG spectrum.

With the uniformity and disorientation of the lattices and the formation of the P_n_V clusters being considered the key factors to the contribution to the Ani-RSHG spectra, the results provide a consistent conclusion that the incorporation of P into a Si ultrathin film degrades the uniformity. This phenomenon can also be observed in the aspect of lattice disorientation. The positive correlation of the $\alpha_{\mathrm{vac}}^{\left(D\right)}$ to the dopant concentration validates the modeling of the SHG polarization at the dopant–vacancy cluster in an analogical manner to the electric dipole moment. The value of *D* serves as an indicator of whether the effect of the dopant on the lattice structure is within the range in which the built model for the SBHM is valid to represent the bond structure.

### 4.1. Effect of Thickness and Calibration

In the actual fabrication, the thickness of the P-doped ultrathin film is hard to be controlled under different growth receipts and expected dopant concentrations. In order to extend the application of our developed SBHM method, an Ani-RSHG spectrum of the PL1 sample with a larger thickness was calibrated through the estimation of the effective thickness for comparison with the P series samples.

**Figure 11:**
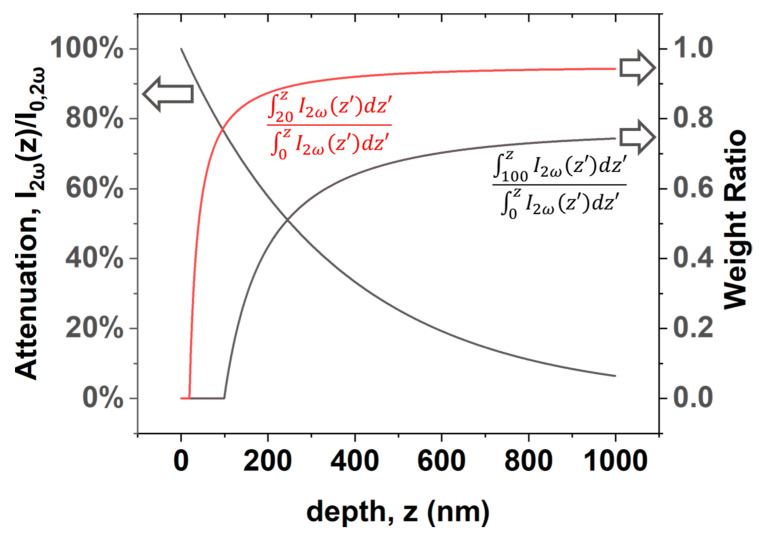
Attenuation and weight ratio of SHG contribution as functions to depth.

Consider the attenuation of the SHG response in Si, such that

(15) $$I_{2\omega }=I_{0,2\omega }e^{-\left(2\alpha_{\omega }d_{\omega }+\alpha_{2\omega }d_{2\omega }\right)},$$

where $I_{2\omega }$ is the attenuated SHG contribution, $I_{0,2\omega }$ is the SHG intensity at the source, $d_{\omega }$ and $d_{2\omega }$ are the traveled distances of the fundamental and SHG lights in the material; $\alpha_{\omega }$ and $\alpha_{2\omega }$ are the absorption coefficients of the fundamental and SHG waves in Si, respectively. With the refractive indices of Si reported in Ref. [42], the angle of incident fundamental and outgoing SHG waves are 11.45° and 9.74° in the Si, respectively. Therefore, Equation (15) can be written as:

(16) $$I_{2\omega }\left(z\right)=I_{0,2\omega }e^{-\left(\frac{2\alpha_{\omega }}{\cos\left(11.45{}^{\circ}\right)}+\frac{\alpha_{2\omega }}{\cos\left(9.74{}^{\circ}\right)}\right)z},$$

where *z* is the depth of the source. Figure 11 shows the profile of the attenuation ratio to the depth of the SHG source, from which the effective depth of the SHG measurement can be found to be approximately 1000 nm, with criteria of 10%. The weight ratio of the contribution from the source in the depth of *z* > 20 nm and *z* > 100 nm are obtained by the integration of Equation (16) and shown in Figure 11. Considering the total contribution from the depth within 1000 nm, the contribution weight ratio of the source in the range of 20~1000 nm and 100~1000 nm is

(17) $$\int_{0}^{1000}I_{2\omega }\left(z\prime \right)dz\prime :\int_{20}^{1000}I_{2\omega }\left(z\prime \right)dz\prime :\int_{100}^{1000}I_{2\omega }\left(z\prime \right)dz\prime =1:0.943:0.743.$$

This ratio implies that one can calibrate the SHG intensity of sample PL1, where the film thickness is 100 nm, for that of a sample with the same P concentration and film thickness of 20 nm by replacing 20% of the contribution of doped Si to that of pure Si, which is the weight ratio of the contribution within the range of depth of 20~100 nm. However, Figure 8 shows that the SHG response of pure Si is far smaller than that of P-doped Si and, thus, neglectable, so the calibration can be conducted by reducing the intensity of sample PL1 by 20%.

### 4.2. Correlation to Fourier Analysis

In the preceding sections, we have established and validated the proposed analysis model based on the SBHM. To recognize the significance of this model, we compare the fitting model based on the SBHM with the Fourier analysis in this section.

The analysis of the Fourier coefficients of the measured Ani-RSHG spectrum is frequently used to describe the symmetrical properties of the structures quantitatively. A common form of Fourier analysis is [26,27]

(18) $$I^{2\omega }\left(\psi \right)={\left|\sum_{m=0}^{4}{\left\{a\right.}^{\left(m\right)}\cos\left[m\left(\psi +\psi_{0}\right)\right]+b^{\left(m\right)}\sin\left[m\left(\psi +\psi_{0}\right)\right]\}\right|}^{2},$$

where $I^{2\omega }$ is the measured SHG intensity, and $\psi_{0}$ is the arbitrary angle wherein the measurement starts. The Fourier coefficient, $C^{\left(m\right)}$, is obtained as

(19) $$C^{\left(m\right)}={\bar{a}}^{\left(m\right)}{}^{2}+{\bar{b}}^{\left(m\right)}{}^{2}$$

(20) $${\bar{a}}^{\left(m\right)}=a^{\left(m\right)}\cos\left(m\psi_{0}\right)+b^{\left(m\right)}\sin\left(m\psi_{0}\right)$$

(21) $${\bar{b}}^{\left(m\right)}=a^{\left(m\right)}\sin\left(m\psi_{0}\right)-b^{\left(m\right)}\cos\left(m\psi_{0}\right)$$

In a homogenous material, clues may be available for some material properties. For example, one can inspect the uniformity of the lattice structure with the variation of C^(4)^ in a Si(100) thin film [43], realize the miscut or macroscopic misorientation with that of C^(1)^ and C^(3)^ [44], or recognize the in-plane anisotropic strain with that of C^(2)^ [44]. However, difficulty has been experienced in interpreting these coefficients in practice due to the contribution of source ambiguity. As a structure becomes more complex through modifications, such as doping, more than one SHG source must be considered. The contributions of these sources to the Fourier coefficient are not isolated, such as C^(1)^ and C^(3)^, which are subject to the magnitude of the vacancy cell dipolar SHG and the tilt of the lattice structure.

By modeling the nonlinear susceptibility tensors of each source independently in the beginning, the fitted parameters can reflect the properties of each source once they obtain different bond structures or atomic compositions. The building of this model provides a theoretical basis for a quantitative analysis that offers a more intuitive interpretation of the result than the conventional Fourier analysis, and thus, this model is promising for identifying more detailed material properties, such as the strain or density of the defects.

## 5. Conclusions

In this study, Ani-RSHG experiments were performed, and the measured spectra exhibited significant difference as the P-doping concentration increased from $2.5\times 10^{17}$ atoms/cm^3^ to $1.6\times 10^{20}$ atoms/cm^3^, demonstrating the high sensitivity of SHG to the incorporation of P dopants into Si ultrathin films. The effects of the photon-induced charging effect and the succeeding EFISHG contribution were evaluated and then prevented by performing an Ani-RSHG measurement under a vacuum of 10^−5^ torr. To analyze the changes in the Ani-RSHG spectra, we proposed a model based on the SBHM to separately predict the SHG contributions from the SiO_2_/Si interface, Si bulk, and P dopant sites in Si. Considering the structure of the P-V clusters, the contributions of the P dopants to Si substitutional sites were modeled with bond vector cells with a vacancy center and P and Si as the neighboring atoms. The contribution of a P dopant site is subject to the atomic arrangement in the cell. The fitted coefficients exhibited a high correlation with the dopant concentration, providing the observations that the lattice uniformity degraded, the lattice disorientation increased, and the SHG contribution from the P dopant sites increased as the doping concentration increased.

Compared with the Fourier analysis conventionally used on Ani-RSHG spectra, this SBHM-based analysis provides a more intuitive interpretation because the coefficients directly correlate to the lattice structure properties. This model exhibits high potential in solving the ambiguity issue experienced when analyzing materials with complex compositions through Fourier analysis. With the assistance of this model, SHG has been demonstrated to be a promising nanophotonic metrology technique for revealing changes in material properties with tens of nm thickness. To achieve the ultimate goal of applying SHG for the in situ real-time monitoring of the film quality or doping concentration, reducing the laser spot size, the time required for measurement, and meeting the vacuum environment to suppress the charging effects are the main challenges to be overcome.

## Data Availability

Not applicable.
